# Understanding the natural language of DNA using encoder–decoder foundation models with byte-level precision

**DOI:** 10.1093/bioadv/vbae117

**Published:** 2024-08-12

**Authors:** Aditya Malusare, Harish Kothandaraman, Dipesh Tamboli, Nadia A Lanman, Vaneet Aggarwal

**Affiliations:** School of Industrial Engineering, Purdue University, West Lafayette, IN 47907, United States; Institute for Cancer Research, Purdue University, West Lafayette, IN 47907, United States; Institute for Cancer Research, Purdue University, West Lafayette, IN 47907, United States; Elmore Family School of Electrical and Computer Engineering, Purdue University, West Lafayette, IN 47907, United States; Institute for Cancer Research, Purdue University, West Lafayette, IN 47907, United States; Department of Comparative Pathobiology, Purdue University, West Lafayette, IN 47907, United States; School of Industrial Engineering, Purdue University, West Lafayette, IN 47907, United States; Institute for Cancer Research, Purdue University, West Lafayette, IN 47907, United States; Elmore Family School of Electrical and Computer Engineering, Purdue University, West Lafayette, IN 47907, United States

## Abstract

**Summary:**

This article presents the Ensemble Nucleotide Byte-level Encoder-Decoder (ENBED) foundation model, analyzing DNA sequences at byte-level precision with an encoder–decoder Transformer architecture. ENBED uses a subquadratic implementation of attention to develop an efficient model capable of sequence-to-sequence transformations, generalizing previous genomic models with encoder-only or decoder-only architectures. We use Masked Language Modeling to pretrain the foundation model using reference genome sequences and apply it in the following downstream tasks: (i) identification of enhancers, promotors, and splice sites, (ii) recognition of sequences containing base call mismatches and insertion/deletion errors, an advantage over tokenization schemes involving multiple base pairs, which lose the ability to analyze with byte-level precision, (iii) identification of biological function annotations of genomic sequences, and (iv) generating mutations of the Influenza virus using the encoder–decoder architecture and validating them against real-world observations. In each of these tasks, we demonstrate significant improvement as compared to the existing state-of-the-art results.

**Availability and implementation:**

The source code used to develop and fine-tune the foundation model has been released on Github (https://github.itap.purdue.edu/Clan-labs/ENBED).

## 1 Introduction

The rise of foundation models in recent years has led to tremendous developments in understanding natural languages (Paaß and Giesselbach 2023). Although they were originally developed to process and generate written text, these models have transcended their initial purpose due to their generalizable nature and wide applicability. Foundation models have shown great potential in the field of bioinformatics (Zhang *et al.* 2023b), since their capacity to be trained on vast amounts of unlabeled data and their adaptability enable them to achieve state-of-the-art performance in a variety of tasks.

Early applications of foundation models in bioinformatics can be seen in analyzing protein sequences (Rives *et al.* 2021, Elnaggar *et al.* 2022), which were then trained on diverse applications like calculation of protein structure, prediction of mutation effects, and the understanding of phylogenetic structure (Lupo *et al.* 2022, Nijkamp *et al.* 2022, Fang *et al.* 2023). These models have since evolved beyond proteins into DNA and RNA analyses, and have demonstrated the ability to surpass previous benchmarks in identifying regulatory elements, predicting chromatin profiles, analyzing evolution from genomic sequence data, and predicting the impacts of mutations in DNA (Ji *et al.* 2021, Yamada and Hamada 2021, Zvyagin *et al.* 2022, Dalla-Torre *et al.* 2023, Nguyen *et al.* 2023). The ability to visualize and interpret the internal model structure (Vig *et al.* 2020) and to derive key insights of the underlying biological processes (Zhang *et al.* 2022) demonstrate the unique advantages offered by foundation models in the field of bioinformatics.

### 1.1 Limitations of previous work

#### 1.1.1 Architecture

Prior work on Transformer-based models for DNA sequence analysis exists in two forms: (i) Encoder-only models (Ji *et al.* 2021, Zhang *et al.* 2022, Fishman *et al.* 2023, Dalla-Torre *et al.* 2023) that focus on classification and regression-based downstream tasks and (ii) Decoder-only models (Nguyen *et al.* 2023, Zhang *et al.* 2023a) that are capable of classification, regression as well as generative tasks that involve design and synthesis.

A combination of encoder and decoder blocks enables the model to perform sequence-to-sequence transformations. One of the fundamental processes undergone by DNA is its transcription into an RNA sequence and subsequent translation into protein sequences, the building blocks of all living organisms. Understanding sequence-to-sequence processes like these is crucial to advancing our knowledge of genetics, and developing an encoder–decoder model is an important step in this direction. Although decoder-only models are capable of sequence-to-sequence transformations, they have no independent means of creating representations of the input sequence, and both input and target tokens are processed in an equivalent fashion. Previous work has shown that a multitask finetuned encoder–decoder Large Language Model outperforms decoder-only models on zero-shot generalization (Sanh *et al.* 2022) as well as targeted tasks like machine translation (Raffel *et al.* 2020, Fu *et al.* 2023). Since a decoder-only architecture will have a unidirectional framework that attends to the source and target sequence simultaneously, as the length of the target sequence grows, the extent to which the model attends to the source will decrease leading to reduced performance in downstream tasks (Fu *et al.* 2023). Our work demonstrates how the cross-attention layers in the decoder leverage the information in the embeddings generated by the encoder, leading to improved performance in training tasks.

#### 1.1.2 Tokenization

Biological sequences like DNA are encoded using a vocabulary of four symbols (A, C, T, G) representing nucleic acids. These sequences are converted into a Transformer-compatible format by a tokenizer, which generates a list of tokens for any given input. Since these models were initially developed for applications in natural languages, the most prevalent forms of tokenization are sentence-piece or word-piece, where the language vocabulary is built using natural ideas like words or syllables. In the absence of typical indicators of linguistic order in DNA, like spaces and punctuation, these tokenization schemes use statistical techniques to determine the “words” that make up the vocabulary of the input sequences. A few examples of previously used tokenizers are: k-mer (Ji *et al.* 2021), SentencePiece (Dalla-Torre *et al.* 2023), and byte-pair encoding (BPE) (Fishman *et al.* 2023) tokenization. While such techniques identify optimal encoding methods by constructing tokens having multiple base pairs, they are vulnerable to any type of noise present in the sequence. A single variation in a base pair will result in the fragment being mapped to a completely different word in the vocabulary, resulting in an outsized impact from a small perturbation (Dotan *et al.* 2024). We use a simplified tokenization scheme where each character corresponds to a single token, resulting in a longer average tokenized length, but more resilience to the variations mentioned above.

### 1.2 Our contributions

In this article, we develop the Ensemble Nucleotide Byte-level Encoder-Decoder (ENBED) Transformer, a foundation model that analyzes nucleotide sequences with Transformers using byte-level tokenization and an encoder–decoder model. This implementation bridges the gap between existing models that are either encoder-only or decoder-only implementations and presents the possibility of sequence-to-sequence analysis tasks. Using sliding-window and global attention we obtain a subquadratic implementation of attention, and demonstrate the performance improvements over dense attention. The foundation model is pretrained using an ensemble of high-quality reference genomes from NCBI RefSeq, including the telomere-to-telomere assemblies of Human and Maize DNA, data from the 1000 Genomes Project and a mix of widely studied organisms like *E. coli*, *D. melanogaster*, *M. musculus*, and *P. vivax* (Sec 6). This process is implemented by giving the model a self-supervised goal of internalizing the structure of the language of nucleotide sequences.

ENBED is built using a byte-level tokenizer. In order to avoid the issues created by single nucleotide variants and their downstream impacts, we side-step the problem of determining the tokenization scheme entirely by working with single nucleotides as tokens. This leads to increased computational costs, but grants resilience to the types of variations and noise commonly encountered in DNA sequences. In order to offset the impact of increased computations, we implement subquadratic attention layers in order to scale up the model efficiently.

#### 1.2.1 Evaluation of performance on genomic benchmark datasets

We evaluate the performance of the ENBED foundation model on sequence-level classification tasks and compare its accuracy against contemporary foundation models. We show that ENBED outperforms the state-of-the-art in 21 of the 25 benchmarks devised by the authors of the Nucleotide Transformer (NT) (Dalla-Torre *et al.* 2023) and Genomic Benchmarks (GB) (Grevsova *et al.* 2022) datasets. These benchmarks consist of tasks like identifying enhancers, promotors, splice sites, and histone marks in multi-species data comprising of genomic sequences from human, mouse, yeast, fruit fly, and worm DNA.

#### 1.2.2 Identifying sequencing noise

Long-read sequencing using Nanopores is used to study telomeres, which are protective caps found at chromosomal ends and have long repetitive elements. It has been found that telomeres in many organisms are frequently miscalled (Tan *et al.* 2022), referring to errors in the process that translates electrical signals into the alphabet of DNA. We illustrate how ENBED can focus on fragments that look incorrect or out of place, demonstrating the model’s ability of distinguishing between noisy and accurate data. In a synthetic dataset constructed using noise distributions found in real-world raw sequence data, we demonstrate that our model can identify sequences containing noise with an accuracy of 97.6%, leveraging the information internalized by bringing pretrained on the telomere-to-telomere reference sequences.

#### 1.2.3 Biological function annotations

Mapping the complete human genome was a significant milestone in modern biology, and it has produced a new set of challenges in identifying the functions and interactions of different parts of the genome. We fine-tune our model to solve a version of this problem by identifying the biological functions of genomic sequences among the most common functional classes using a fine-tuned model, achieving an *F*_1_ score of 74.1.

#### 1.2.4 Studying mutations as a sequence-to-sequence process

Exploring mutations is essential as it sheds light on the mechanisms driving genetic diversity which enhance the overall resilience of living organisms in a changing environment. The encoder–decoder architecture confers the ability to rapidly iterate mutagenization of genomic segments. We study mutations in the Influenza virus, using the NCBI Influenza Virus Resource. By constructing a dataset with a phylogenetic tree, we obtain parent–child pairs of mutated sequences and show the effectiveness of our encoder–decoder architecture in analyzing and predicting these mutations.

## 2 Methods

### 2.1 Encoder–decoder model architecture

**Figure 1.**
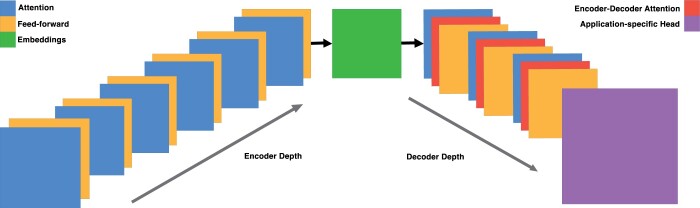
Model architecture. The model is constructed using encoder and decoder blocks with a ratio of 2:1. Both types of blocks consist of attention and feed-forward layers, with the decoder blocks additionally incorporating the embeddings in encoder–decoder attention layers.

ENBED is built using an encoder–decoder architecture (Fig. 1) consisting of encoder and decoder blocks, each comprised two subcomponents: An attention layer and a feed-forward neural network. The attention layers process a sequence by replacing each element with a weighted sum of linear transformations of the input embeddings, after which they are normalized and passed through the feed-forward neural network. Dropout is applied to the feed-forward network, the attention weights, and the input and output of the entire stack. The implementation is written using JAX (Bradbury *et al.* 2018) and the Flax-former library (Heek *et al.* 2023).

We formulate a model with 1.2B trainable parameters, with the configuration specified in Table 6. The model is encoder-heavy since idiosyncratic relationships among tokens are better encoded by devoting a larger share of parameters to these blocks. We found that adjusting the encoder-to-decoder ratio to 2:1 improved performance, with a 1% increase in Masked Language Modeling (MLM) accuracy for all model sizes over the 3:1 ratio chosen by the authors of ByT5 (Xue *et al.* 2022), a similar architecture built to process token-free text-to-text transformations. We also find that reducing the masked span length, which is the average number of tokens masked during pretraining, from 40 down to 20 helps in faster convergence owing to the significantly smaller vocabulary of DNA.

**Table 6.** Model configurations.

Configuration	Decoder-only (no cross-attn.)	Base model 1:1 Enc/Dec	ENBED
Parameters	800M	800M	1.2B
*d*_ff_	3584	3584	3850
*d*_kv_	64	64	64
*d*_model_	1536	1536	1536
Encoder layers	0	12	24
Decoder layers	24	12	12
Attention heads	16	16	16
Global attention (*k*)	128	128	256
Top-1 accuracy (%)	53.1	62.0	76.9

*d*_model_ denotes the size of the encoder layers, and the pooler layer, *d*_kv_ is the size of the key, query, and value projections per attention head and *d*_ff_ is the size of the intermediate feed-forward layer in each Transformer block. The accuracy of the top-1 candidate is evaluated using the same framework used in Table 5.

### 2.2 Tokenization

Sequences are tokenized by breaking down the input into tokens consisting of single nucleotides. The vocabulary size is fixed at 384, with 256 ASCII characters and additional tokens added to function as MASK, PAD, and UNKNOWN tokens during the training process. We require multiple MASK tokens in order to index the positions where masking has occurred and to label the targets with these indices. Although the alphabet of DNA only comprises the four nucleic acids Adenine (A), Cytosine (C), Guanine (G), and Thymine (T), we choose to keep the whole set of extended ASCII characters since they could aid in future tasks like sequence-to-sequence transformations involving targets beyond just DNA sequences, like drug structures represented by the SMILES notation system.

This approach requires more floating-point operations as compared to other tokenization methods, since it increases the tokenized sequence length for the same input DNA sequences, resulting in higher resource requirements. Although this limits us to dealing with short- to medium-length sequences, we can overcome these constraints and scale up the model by reducing the complexity of attention layers as described below.

### 2.3 Attention

Attention can be understood as a soft-lookup of a query **Q** in a dictionary of stored keys **K** and values **V**. Attention scores are generated by calculating the similarity between **Q** and **K**, each having a dimension *d*, with scaled dot-product attention $\left(\text{Softmax}\left(QK^{T}/\sqrt{d}\right)V\right)$ being the most common implementation. Increasing the sequence length *L* can be a challenge, since this type of attention has a complexity of $O(L^{2})$. This sets a limit of L≤512 tokens on our hardware *(*NVIDIA A100 (40 GB) GPUs).

In order to reduce the complexity while preserving function, we modify the architecture to replace dense attention with a combination of two subquadratic variants of attention: (i) sliding-window attention and (ii) global attention.

#### 2.3.1 Sliding-window attention

Local context is crucial in analyzing DNA, since biological processes like transcription and translation work within continuous regions of a sequence. Tokens within a sliding window of radius *r* are used to calculate the attention scores, bringing the complexity down to O(L×r). We fix *r*** **=** **64 for the initial three layers and increase to *r*** **=** **128 in the final layers, which allows them to learn higher-level representations while having the lower layers focus on local information.

#### 2.3.2 Global attention

For tasks that involve classifying or annotating whole sequences, we need a mechanism that aggregates global information from the inputs, in addition to the local scores. We divide the input sequence into *k* blocks and calculate a global token by summing and normalizing the embeddings for every token in the block. Scores are then computed for every input token by letting it attend to the neighboring tokens (as described above) and all the global tokens, which has a total complexity of O(L(r+k)).

Hence, by choosing appropriate values for *r* and *k* relative to *L*, we implement a scheme to calculate attention with a subquadratic complexity which allows us to set an input and output length of 16 384, a significant improvement over the limit of 512 tokens using dense attention with the same GPU hardware.

The aggregated blocks constructed in this procedure resemble previous tokenization schemes like k-mer, used by previous models like DNABERT (Ji *et al.* 2021) and BPE used by GENA-LM (Fishman *et al.* 2023). Our method uses a combination of these aggregated blocks along with higher-granularity local context to achieve a balance between the two, allowing us to process sequences with greater precision.

### 2.4 Applications of foundation models using transfer learning

#### 2.4.1 Building the foundation model

The first step in building our foundation model is pretraining it on high-quality reference sequences. We use a procedure called MLM. The objective is to reconstruct tokens that have been deleted and replaced with a MASK token. This task develops the ability to understand the context and vocabulary to identify the correct elements that belong in the masked segments. Utilizing a large corpus of unlabeled data allows us to impart the model with generalizable knowledge that can be fine-tuned for specific downstream tasks. The genomic corpus is constructed by concatenating FASTA files from the NCBI sources mentioned in the Data Availability section, removing any descriptions starting with “>” and “N” bases that are a result of hard-masking. We choose a masking rate of 15% over the course of pretraining. The entire corpus is supplied to a collator that handles masking, padding, and truncation to ensure that the input length is maintained. We follow a linear schedule with warmup (5% of the total training steps) using the AdamW optimizer ($\beta_{1}=0.9,\beta_{2}=0.99,\epsilon =10^{-6}$) with a learning rate of 1e-5, a cross-entropy loss function and softmax as the activation function. We train all versions of the model with maximum input and output lengths of 16,384 tokens (base pairs). Convergence takes 120–480 GPU-hours with 8 NVIDIA A100 GPUs, determined by model size and input length.

#### 2.4.2 Fine-tuning for downstream tasks

We fine-tune the model by modifying the final layers into a task-specific configuration. This is called the “head” of the model and is attached to the final layer of the pretrained model. Layers are gradually unfrozen in reverse order during the course of fine-tuning, allowing the Transformer to integrate with the attached head while retaining the initial layers, thus enabling the transfer of pretrained knowledge for downstream applications.

#### 2.4.3 Classification head

A fully connected (dense) layer is usually added to the output of the base model, followed by a softmax activation to produce class probabilities, typically used in sequence-level classification tasks.

#### 2.4.4 Language modeling head

A language modeling head comprises a single feedforward neural network layer followed by a softmax activation function. This layer takes hidden representations from the preceding layers and outputs a probability distribution over the vocabulary. The objective is to estimate the estimate the probability of a token given the previous words in a sentence. The softmax function transforms the raw output scores into probabilities, representing the likelihood of each word or token in the vocabulary at any particular position. This process is called autoregressive generation, and we use it to perform sequence-to-sequence transformations.

### 2.5 Application domains

The ENBED foundation model is evaluated across a set of genomic analysis tasks to demonstrate its versatility and the unique advantages of its encoder–decoder architecture. We begin with the GB and NT Benchmarks, which provide standardized comparisons against existing models for fundamental sequence classification tasks. The noise identification task assesses ENBED’s ability to distinguish genuine sequences from artifacts, leveraging its byte-level precision. Biological function annotation tests the model’s capacity to associate sequence patterns with higher-level functions, crucial for genome interpretation. Finally, the mutation generation task is an end-to-end evaluation of the ENBED, a novel architecture not present in previous genomic language models. This sequence-to-sequence task, focused on predicting viral mutations, showcases ENBED’s potential for modeling complex genomic transformations.

#### 2.5.1 Genomic benchmarks

The GB dataset consists of sequences from four organisms: Human, mouse (*Mus musculus*), roundworm (*Caenorhabditis elegans*), and fruit fly (*Drosophila melanogaster*). The dataset comprises: (i) Human enhancers from Cohn *et al.* (2018) and Ensembl (Martin *et al.* 2022), (ii) Open Chromatin Region classifications from the Ensembl build, (iii) Computationally generated data for coding and noncoding sequences (iv) Multi-class data composed of three regulatory elements (promotors, enhancers, and Open Chromatin Regions), (v) Non-TATA promotor sequences imported from Umarov and Solovyev (2017).

#### 2.5.2 Nucleotide transformer benchmarks

The NT benchmarks consist of five data sources: (i) Epigenetic marks in the yeast genome, which use experimentally obtained nucleosome occupancy values processed into positive and negative observations and to provide the following histone marks datasets: {H3, H4, H3K9ac, H3K14ac, H4ac, H3K4me1, H3K4me2, H3K4me3, H3K36me3, and H3K79me3}, (ii) A dataset (Geng *et al.* 2022) consisting of a mix of strong, weak, and nonenhancers. (iii) Promotor sequences 300 base pairs in length around transcription start sites, divided on the basis of TATA and non-TATA box promotors. (iv) Splice site datasets composed of donor, acceptor, and nonsplice site sequences from phylogenetically diverse organisms.

#### 2.5.3 Noise identification

We generate a synthetic dataset with segments of 512 nucleotides selected at random from TeloBase (Lyčka *et al.* 2023), a comprehensive database of information about telomere motif diversity. We introduce noise based on real-world raw DNA sequencing data to generate negative samples. Previous work (Rabadan *et al.* 2017) finds that noise in sufficiently deep DNA sequencing data can be approximated by aggregating negative binomial distributions. Using this method, we create a balanced dataset with positive and negative samples. The model is fine-tuned on a sequence classification task with this labeled dataset. This process can be likened to out-of-distribution detection (Fort *et al.* 2021), since the negative samples would represent data that does not belong to the distribution of the training dataset. We describe this procedure in more detail in the Supplementary Material (Section B).

#### 2.5.4 Biological function annotation

We can formulate the process of annotating genes as a classification task, with the input being a DNA sequence fragment and the output being the class probabilities for the annotation types defined below. For evaluating our model, we train it to output the biological function annotation of a given genomic input sequence up to 512 base pairs in length. We choose the following annotation types for our experiment: Coding Sequences, IncRNA, snoRNA, miscRNA, miRNA, snRNA, TEC, Processed, and Unprocessed Pseudogenes. These annotations are obtained from the Ensembl dataset (Martin *et al.* 2022), and the constructed dataset has an equal number of examples for all classes. We generate 9216 training examples and 1024 validation examples for this task.

#### 2.5.5 Mutation generation

Human influenza A viruses are named based on the geographic location where the virus was isolated, the date of the isolate, and the identity of the two major surface proteins, hemagglutinin (HA) and neuraminidase. We choose the HA1 sequences to create the Influenza virus mutation dataset, selecting the segments with the most highly variable regions for training and validation. We obtain our source data from Berman *et al.* (2023) and subset the HA1 nucleotide sequence of the H3N2 Influenza virus between 300 and 799 bp (100–266 amino acids) to capture the Antigenic site A and B. The selected region is a part of the globular domain that occurs in a jelly-roll fold of eight-stranded antiparallel beta-sheets, containing the most commonly mutating amino-acid residues around the receptor binding site. The HA1 head also accumulates N-linked glycosylation sites over time, which are thought to mask antigenic sites from immune recognition. The glycosylation of the HA1 globular domain modulates receptor binding, stimulates host antibody responses, and shields key antigenic sites to facilitate immune evasion of the virus. By focusing on the HA1 subdomain, we aimed to evaluate the sequence-to-sequence model on a functionally important region of influenza HA that experiences significant antigenic drift and glycosylation changes. The Supplementary Material contains additional details about the construction of training and validation splits for the dataset.

Candidate sequences are generated using a language modeling head with the parent sequence supplied as the input. Using a beam search (*N*_beams_ = 5), we obtain five candidate sequences which are autoregressively generated to a length of 499 bp (equal to the input). We rank the sequences using the noise identification pipeline above, and select the sequence least likely to be identified as having noise present. We identify mutations by measuring the Levenshtein distance between parent and child sequences. This metric accounts for insertion, deletion as well as in-place modifications.

## 3 Results

**Figure 2.**
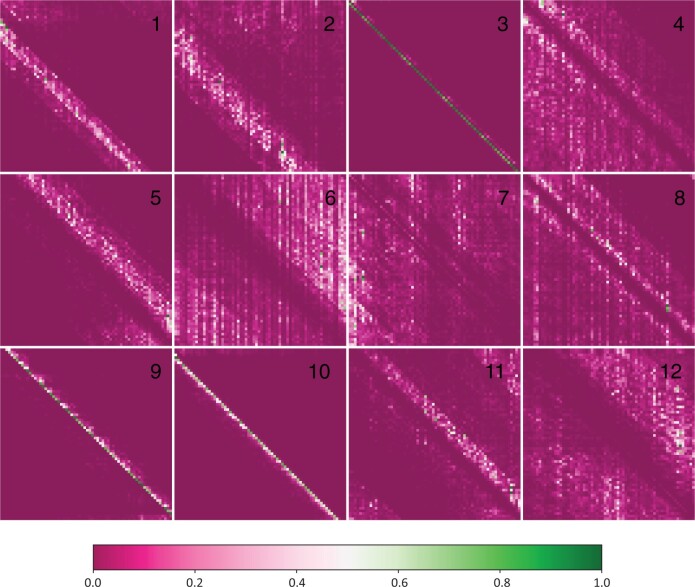
Interpreting attention layers. We visualize the twelve attention heads of the pretrained ENBED foundation model.

Upon convergence, the pretraining process yields a foundation model ready to be applied to downstream tasks. The initial layers in the pretrained model are frozen since they contain generalizable information that helps the model build versatile internal representations of the data. We visualize these internal representations by extracting the encoder output layer and plotting attention maps in Fig. 2. These maps are generated using the outputs from the final encoder block. The use of multiple attention heads grants the model the ability to simultaneously use a diverse range of patterns to analyze input sequences. In Fig. 2, we observe that some heads are dedicated to analyzing close neighbors (3, 9, 10) while others display a more dilated version of this phenomenon (1, 2, 5, 11). Additionally, there are heads which attempt to exclude local information and focus on a more global view of the input sequence (4, 6, 8, 12).

### 3.1 ENBED outperforms state-of-the-art models on GB datasets

We finetune the model using a classification head using the embedding outputs from the final encoder block, on the datasets constructed by the authors of the NT benchmarks (Dalla-Torre *et al.* 2023) and GB (Grevsova *et al.* 2022). The results of evaluating the model on the test dataset of NT and GB are presented in Tables 1 and 2, respectively. For evaluation of the NT benchmarks, we compare our performance against the NT (v2) and HyenaDNA (Dalla-Torre *et al.* 2023, Nguyen *et al.* 2023), which are encoder-only and decoder-only models, respectively. For the GB datasets, we use the performance of the Convolutional Neural Network (CNN) model developed by the authors of the dataset (Grevsova *et al.* 2022) as a baseline. We also include the performance of the HyenaDNA model and the baseline Transformer developed by its authors (Nguyen *et al.* 2023).

**Table 1.** Nucleotide transformer (NT) benchmarks.

NT benchmark	Enformer	DNABERT-2	NT (2.5B)	HyenaDNA (1 kb)	ENBED (GRCh38)	ENBED
H3	0.719	0.785	0.791	0.779	0.723	**0.802**
H3K14ac	0.288	0.516	0.537	0.612	0.537	**0.636**
H3K36me3	0.344	0.591	0.616	0.613	0.611	**0.624**
H3K4me1	0.291	0.511	0.544	0.512	0.498	**0.591**
H3K4me2	0.211	0.336	0.322	0.455	0.433	**0.501**
H3K4me3	0.158	0.352	0.408	0.549	0.580	**0.587**
H3K79me3	0.496	0.613	0.621	0.672	0.648	**0.756**
H3K9ac	0.420	0.542	0.550	0.581	0.427	**0.590**
H4	0.732	0.796	0.807	0.763	0.750	**0.823**
H4ac	0.273	0.463	0.489	0.564	0.548	**0.605**
Promotor (all)	0.909	0.943	0.950	0.920	0.906	**0.961**
Promotor (non-TATA)	0.909	0.944	0.952	0.921	0.892	**0.959**
Promotor (TATA)	0.920	0.910	0.919	0.882	0.883	**0.944**
Splice acceptor	0.829	0.950	**0.973**	0.915	0.754	0.943
Splice donor	0.814	0.926	**0.974**	0.898	0.835	0.911
Enhancer	0.451	0.516	0.548	0.517	0.577	**0.585**
Enhancer Types	0.309	0.423	0.450	0.386	0.459	**0.482**

We evaluate our model using the 10-fold mean Matthews Correlation Coefficient (MCC) of the best performing variants of the Enformer (Avsec *et al.* 2021), DNABERT (Zhou *et al.* 2024), Nucleotide Transformer v2 (Dalla-Torre *et al.* 2023), and HyenaDNA (Nguyen *et al.* 2023), highlighting the **best** and second-best scores. The scores are sourced from a leaderboard maintained by the authors of Dalla-Torre *et al.* (2023) on the Hugging Face platform (InstaDeepAI 2023).

**Table 2.** Genomic benchmarks.

Genomic benchmark	CNN	DNABERT	GPT	**HyenaDNA** (Nguyen *et al.* 2023 **)**	ENBED (GRCh38)	ENBED
Mouse Enhancers	69.0	66.9	80.1	85.1	81.1	**90.3**
Human Enhancers (Cohn)	69.5	74.0	70.5	**74.2**	70.8	71.2
Human Enhancers (Ensembl)	68.9	85.7	83.5	89.2	90.2	**92.2**
Coding versus Intergenomic	87.6	92.5	88.8	91.3	90.7	**93.0**
Human versus Worm	93.0	96.5	95.6	96.6	94.4	**97.3**
Human Regulatory Elements	93.3	88.1	91.5	**93.8**	85.6	90.2
Human Promoter (Non-TATA)	84.6	85.6	87.7	96.6	90.4	**97.2**
Human OCR (Ensembl)	68.0	75.1	73.0	80.9	76.2	**81.9**

Accuracy (%) scores of the **best** and second-best model in the Genomic Benchmarks datasets (Grevsova *et al.* 2022). The baseline CNN and GPT scores were calculated by the authors of Grevsova *et al.* (2022) and Nguyen *et al.* (2023), respectively.

ENBED demonstrates superior performance, exceeding state-of-the-art results in 15 out of 17 NT benchmarks and 6 out of 8 GB datasets. This improvement likely stems from our novel approach combining byte-level analysis, high-quality reference sequences, and an optimized pretraining methodology. We hypothesize that byte-level tokenization enhances the model’s ability to handle variations such as single nucleotide polymorphisms, while our encoder–decoder architecture enables simultaneous focus on multiple input sections and context-aware processing. These features may contribute to ENBED’s advantages over decoder-only methods. While the relative impact of each component requires further investigation through ablation studies, our results demonstrate ENBED’s effectiveness across a wide range of genomic analysis tasks.

### 3.2 ENBED identifies noise in genomic sequences

Table 3 shows the results of the sequence-level classification of erroneous sequences using our synthetic dataset. Since competing models are trained using the GRCh38 reference assembly, they often lack information about repetitive regions due to hard-masking. Our choice of higher-quality pretraining data results in a significant performance improvement and an overall accuracy of 97.1% in the sequence-level classification task of identifying erroneous genomic data, which is a significant improvement as compared to the baselines of DNABERT (Ji *et al.* 2021) (84.9%) and NT (Dalla-Torre *et al.* 2023) (91.8%).

**Table 3.** Erroneous sequence identification.

Model	Reference	*F*_1_ score
DNABERT	(Ji *et al.* 2021)	84.9
Nucleotide Transformer	(Dalla-Torre *et al.* 2023)	91.8
**ENBED**	This article	**97.6**

### 3.3 ENBED identifies biological function annotations

ENBED is trained to identify the annotations (defined in Section 2.5) of the Human reference assembly. As shown in Table 4, we achieved an *F*_1_ score of 74.1 in this classification task, an improved score compared to DNABERT (Ji *et al.* 2021) (63.2), NT (Dalla-Torre *et al.* 2023) (67.5), and HyenaDNA (Nguyen *et al.* 2023) (72.8). For the purposes of this evaluation, all models were finetuned and evaluated using the same balanced dataset as specified in Section 2.5.

**Table 4.** Biological function identification.

Model	Reference	*F*_1_ score
DNABERT	(Ji *et al.* 2021)	63.2
Nucleotide Transformer	(Dalla-Torre *et al.* 2023)	67.5
HyenaDNA	(Nguyen *et al.* 2023)	72.8
**ENBED**	This article	**74.1**

**Table 5.** Mutation generation.

Model	Top-1 accuracy	Top-5 accuracy	Mean LD	Median LD
Transformer (BPE tokenization)	32.0	56.1	30.6	24
ENBED (decoder-only)	53.1	72.1	6.1	4
ENBED	**76.9**	**95.4**	2.3	1

Accuracy (%) scores of Top-1 and Top-5 candidates with the mean and median Levenshtein Distance (LD) between predicted and child sequences.

### 3.4 ENBED generates mutations using sequence-to-sequence transformation

We evaluate the accuracy of ENBED in generating mutations, using an encoder–decoder Transformer with BPE tokenization *(*used in previous genomic models; Fishman *et al.* 2023) as a baseline. We compare against BPE because this method shares similarities with byte-level tokenization by starting with the basic {A, C, T, G} alphabet, but tries to optimize the vocabulary by combining simpler words into more complex ones based on the corpus the tokenizer is trained on. The training corpus itself is identical to the one used in pretraining ENBED, with the only difference being the tokenization procedure. While this procedure reduces the average number of tokens generated from any input sequence, it also results in reduced accuracy since modifying even a single base pair will output a significantly different tokenized sequence.

Top-1 and Top-5 Accuracy (%) scores are calculated by comparing predictions with real-world data from the Influenza Virus Resource (Bao *et al.* 2008), with any deviation from an exact match being classified as incorrect. Top-5 scores are calculated by selecting the best candidate from the procedure described in Sec 2.5. Additionally, we also train a version of ENBED with the encoder removed, as a comparison of the sequence-to-sequence task performance between decoder-only and encoder–decoder models.

The mean Levenshtein distance of our model predictions from real-world mutated sequences is 2.3 edits over a length of 500 bp, resulting in an average similarity of 99.5%. We can attribute the significant increase in accuracy to byte-level tokenization, since other schemes with tokens involving multiple base pairs will be unable to capture edits involving single nucleotides effectively.

## 4 Ablation studies

We perform ablation studies in order to examine the impact of the architectural modifications and the combination of encoder and decoder blocks.

### 4.1 Encoder–decoder architecture

We study the impact of combining encoder and decoder blocks and the cross-attention links between them in Table 6. A decoder-only version of the model is constructed by stacking 24 decoder layers and is pretrained to convergence using next-token prediction. We also construct a balanced model using stacks of 12 layers for both the encoder and decoder blocks, introducing cross-attention layers in the decoder that attend to the embeddings and the output sequence. Both models have ∼ 800 M trainable parameters. We then fine-tune these models on the mutation generation task and compare with the ENBED model having a 2:1 encoder–decoder block ratio.

Introducing the encoder and cross-attention leads to a significant improvement in the pretraining accuracy, demonstrating the suitability of both the architecture as well as the pretraining task, since decoder-only models are restricted to causal objectives like next-token prediction unlike encoders that can handle bidirectional information.

## 5 Discussion

The ENBED model demonstrates significant improvements over existing approaches in several areas of genomic sequence analysis. The encoder–decoder architecture, combined with byte-level tokenization and high-quality pretraining data, contributes to enhanced performance across multiple tasks. ENBED’s performance on established benchmarks is noteworthy, surpassing state-of-the-art results in 21 out of 25 tasks across the NT and GB datasets. This broad improvement suggests that our approach captures underlying genomic patterns more effectively than previous models. Additionally, the model successfully identified sequences containing noise with an accuracy of 97.6%, demonstrating its sensitivity to small-scale genomic perturbations. This is likely due to the byte-level tokenization approach used in ENBED, which allowed for the accurate detection of variations at single-nucleotide resolutions.

The encoder–decoder structure proves particularly effective for sequence-to-sequence tasks like mutation generation. Our results show that ENBED outperforms baseline models in predicting Influenza virus mutations, achieving a top-5 accuracy of 95.4%. This was a significant improvement over the baseline model using BPE tokenization (56.1%), and another variant of ENBED without the encoder (72.1%). We chose to vary both the tokenization scheme and architecture in these cases while keeping the rest of the design choices unchanged in order to isolate the impact of these two factors. We find that the choice of BPE tokenization significantly impacts the model’s ability to generate mutations accurately, with byte-level tokenization providing a clear advantage due to its ability to capture single-nucleotide changes. We also see that an encoder–decoder architecture is crucial for this task, as the decoder-only model does not perform as well, following the trend observed in other sequence-to-sequence tasks (Raffel *et al.* 2020, Fu *et al.* 2023).

It is also worth noting that the use of higher-quality pretraining data, including telomere-to-telomere assemblies, may contribute to ENBED’s improved performance. This comprehensive genomic representation likely allows the model to learn from previously underrepresented genomic regions. A study of the NT benchmarks (Table 1) with two versions of ENBED trained on different reference assemblies (GRCh38 and T2T-CHM13) showed that the model trained on the higher-quality T2T-CHM13 assembly outperformed the GRCh38 model across the board. This suggests that the choice of reference assembly can significantly impact the model’s performance, and that the use of more complete and accurate reference genomes can lead to better generalization.

Future work on this model could explore additional applications in genomics, such as variant effect prediction and protein structure studies.

## Supplementary Material

vbae117_Supplementary_Data

## Data Availability

The telomere-to-telomere reference sequences for Human (GCF_009914755.1) and Maize (GCA_022117705.1) and the reference sequences for *E. coli* (GCF_000008865.2), *D. melanogaster* (GCF_000001215.4), *M. musculus* (GCF_000001635.27) and *P. vivax* (GCF_000002415.2) were obtained from NCBI RefSeq (O’Leary *et al.* 2016) in FASTA format. Variant Calling Files (VCFs) for the 1000 Genomes Project (1000 Genomes Project Consortium 2015) were obtained from the European Bioinformatics Institute. Gene annotations were obtained from GENCODE (Harrow *et al.* 2012) and Ensembl (Martin *et al.* 2022). The mutation tree was derived from the data assembled by the authors of Berman *et al.* (2023), sourced from the NCBI’s Influenza Virus Resource (Bao *et al.* 2008). The source code used to develop and fine-tune the foundation model has been released on Github (https://github.itap.purdue.edu/Clan-labs/ENBED) and the weights of the model used in evaluation are available here (https://huggingface.co/malusare).
