# Supplementary material for: Understanding the natural language of DNA using encoder–decoder foundation models with byte-level precision
Source: Bioinform Adv. 2024 Aug 12;4(1):vbae117. doi: 10.1093/bioadv/vbae117 (PMC11341122; doi:10.1093/bioadv/vbae117)
Supplement: vbae117_Supplementary_Data [file vbae117_supplementary_data.pdf]

Supplementary Material

A Pre-training Data Sources

Table 1 shows the pre-training data sources used for the Enformer [Avsec et al., 2021], DNABERT-2 [Zhou et al., 2023], Nucleotide Transformer v2 [Dalla-Torre et al., 2023], and HyenaDNA [Nguyen et al., 2023] models. We also construct a GRCh38-based version of ENBED as mentioned in Tables 1 and 2 in the main paper.

B Task-specific Datasets

B.1 Nucleotide Transformer

For epigenetic marks prediction, a dataset of acetylation and methylation nucleosome occupancies in the yeast genome was used, with data from Chip-Chip experiments processed into positive and negative observations for 10 histone marks. Promoter sequence prediction utilized a dataset of 29,597 promoter regions, including 3,065 TATA-box promoters, with sequences spanning 300bp around transcription start sites. Matched negative samples were created by shuffling promoter sub-sequences.

Enhancer sequence prediction relied on a single dataset that originally contained 742 strong, 742 weak, and 1484 non-enhancers, which was augmented with 6000 synthetic enhancers and 6000 synthetic non-enhancers to evaluate the transformer's representation of enhancers. Splice site prediction employed two datasets: the SpliceFinder dataset, which included donor, acceptor, and non-splice sites in human genes with 400bp sequences, and the Spliceator training set, which consisted of 600bp sequences from diverse organisms, using a balanced 'Gold Standard' subset.

Table 2, sourced from Dalla-Torre et al. [2], shows the dataset statistics for the various genomic sequence classification tasks.

B.2 Genomic Benchmarks

The Genomic Benchmarks dataset consists of 8 classification tasks, each with a unique set of positive and negative sequences. The tasks include the classification of mouse enhancers, human enhancers (Cohn), human enhancers (Ensembl), coding vs. intergenic regions, human vs. worm, human regulatory elements, human promoters (non-TATA), and human OCR (Ensembl). The dataset is designed to evaluate the performance

**Model**	**Data Source**	**Description**
Enformer [Avsec et al., 2021]	GRCh38 + GRCm38	Human and Mouse reference genomes
DNABERT-2 [Zhou et al., 2023]	GRCh38 + Multi-species Dataset	Multi-species data consists of 135 species randomly selected across 7 categories.
Nucleotide Transformer [Dalla-Torre et al., 2023]	GRCh38 + 1000G + Multi-species Dataset	Versions with the Human reference genome, 1000 Genomes project (1000G), and multi-species data consists of 850 species.
HyenaDNA [Nguyen et al., 2023]	GRCh38	Human reference genome

Table 1: **Pre-training Data Sources.**

	Num train sequences	Num test sequences	Max sequence length in bp
H3K4me3	25953	2884	500
H3K4me2	27614	3069	500
H3K36me3	31392	3488	500
H3K9ac	25003	2779	500
Splice donor	19775	2198	600
Splice site all	27000	3000	400
H4ac	30685	3410	500
H3K4me1	28509	3168	500
Enhancer	14968	400	200
Enhancer types	14968	400	200
H4	13140	1461	500
Splice acceptor	19961	2218	600
H3K79me3	25953	2884	500
Promoter non-TATA	47767	5299	300
Promoter all	53276	5920	300
H3K14ac	29743	3305	500
H3	13468	1497	500
Promoter TATA	5509	621	300

Table 2: Dataset statistics for Nucleotide Transformer classification tasks

of models on a diverse set of genomic sequence classification tasks. Table 3 shows the dataset statistics for the Genomic Benchmarks tasks.

B.3 Noise Generation

We generated a synthetic dataset to evaluate our model's capacity to differentiate between genuine sequences and those containing errors. The dataset was constructed using segments of 512 nucleotides selected at random from TeloBase, a comprehensive database of telomere motif diversity.

Noise was injected as per the distribution found in the work of [Rabadan et al., 2017] using a deepSNV-based implementation [Gerstung et al., 2012]. The dataset was divided into training and test sets with 10,000 and 1,000 sequences, respectively.

B.4 Mutation Generation

For the mutation generation task, we employ a fine-tuning approach using a sequence-to-sequence model. This model is trained to predict child sequences given parent sequences, effectively learning the patterns of mutations observed in the influenza virus population. To ensure the robustness of our results and prevent overfitting, we have implemented a comprehensive strategy for constructing our training and test datasets.

Our approach begins with the construction of a phylogenetic tree from the available influenza virus sequences using a maximum likelihood method. Figure 1 shows a circular cladogram visualization of the generated Influzenza H1 gene sequences, where nodes are represented by yellow dots. This tree provides a representation of the evolutionary relationships between different strains. We use this phylogenetic information to inform our data split, ensuring that closely related strains are not separated between the training and test sets. Specifically, we implement a monophyletic clade-based splitting strategy, where entire clades below a certain depth in the tree are assigned to either the training or test set. This step is crucial to prevent information leakage and maintain the integrity of our evaluation.

Furthermore, we implement a sequence similarity cutoff of 95% using the Levenshtein distance metric to address the issue of high sequence homology between training and test sets. Sequences with greater than 95% similarity are grouped together and assigned entirely to either the training or test set, never split between the two. In total, we create 5000 parent-child sequence pairs for training and 500 pairs for testing.

Name	# of sequences	# of classes	Class ratio	Median length	σ
dummy_mouse_enhancers_ensembl	1210	2	1.0	2381	984.4
demo_coding_vs_intergenomic_seqs	100000	2	1.0	200	0.0
demo_human_or_worm	100000	2	1.0	200	0.0
drosophila_enhancers_stark	6914	2	1.0	2142	285.5
human_enhancers_cohn	27791	2	1.0	500	0.0
human_enhancers_ensembl	154842	2	1.0	269	122.6
human_ensembl_regulatory	289061	3	1.2	401	184.3
human_nontata_promoters	36131	2	1.2	251	0.0
human_ocr_ensembl	174756	2	1.0	315	108.1

Table 3: Description of datasets in genomic benchmark package. Name is the unique identification of dataset. # of sequences is the combined count of all sequences from all classes. # of classes is the count of all classes in a dataset. Class ratio is the ratio between number of sequences in the largest and smallest classes. Median length and Standard deviation are computed for all sequences from all classes in a dataset. (Reproduced from [Grevsova et al., 2022])

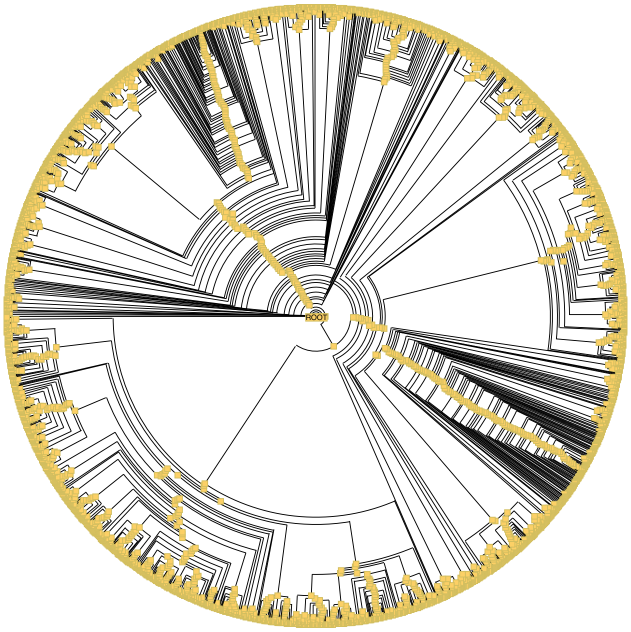

Figure 1: **Phylogenetic Tree.**

NT Benchmark	Peer-reviewed Baselines	ENBED (GRCh38)	ENBED	Std. Dev.
H3	0.791 [Dalla-Torre et al., 2023]	0.723	0.802	0.031
H3K14ac	0.612 [Nguyen et al., 2023]	0.537	0.636	0.020
H3K36me3	0.616 [Dalla-Torre et al., 2023]	0.611	0.624	0.016
H3K4me1	0.544 [Dalla-Torre et al., 2023]	0.498	0.591	0.009
H3K4me2	0.455 [Nguyen et al., 2023]	0.433	0.501	0.035
H3K4me3	0.549 [Nguyen et al., 2023]	0.580	0.587	0.018
H3K79me3	0.672 [Nguyen et al., 2023]	0.648	0.756	0.014
H3K9ac	0.581 [Nguyen et al., 2023]	0.427	0.590	0.006
H4	0.807 [Dalla-Torre et al., 2023]	0.750	0.823	0.011
H4ac	0.564 [Nguyen et al., 2023]	0.548	0.605	0.017
Promotor (all)	0.950 [Dalla-Torre et al., 2023]	0.906	0.961	0.021
Promotor (non-TATA)	0.952 [Dalla-Torre et al., 2023]	0.892	0.959	0.019
Promotor (TATA)	0.920 [Avsec et al., 2021]	0.883	0.944	0.017
Splice acceptor	0.973 [Dalla-Torre et al., 2023]	0.754	0.943	0.034
Splice donor	0.974 [Dalla-Torre et al., 2023]	0.835	0.911	0.029
Enhancer	0.548 [Dalla-Torre et al., 2023]	0.577	0.585	0.011
Enhancer Types	0.450 [Dalla-Torre et al., 2023]	0.459	0.482	0.007

Table 4: **Nucleotide Transformer (NT) Variances.**

C Variances for the Nucleotide Transformer Benchmarks

Table 4 shows the standard deviations of the 10-fold Matthews Correlation Coefficient (MCC) scores for the Nucleotide Transformer (NT) benchmarks. The peer-reviewed baselines are sourced from a leaderboard maintained by the authors of [Dalla-Torre et al., 2023] on the Hugging Face platform [InstaDeepAI, 2023].

D Evaluation Metrics

D.1 Matthew's Correlation Coefficient

The Matthews Correlation Coefficient (MCC), originally introduced by Matthews in 1975 for binary classification, has been extended to multi-class classification scenarios [?]. In the multi-class context, the MCC provides a balanced measure of the quality of classification that is particularly useful when dealing with imbalanced datasets. For a classification problem with K classes, the multi-class MCC is defined as:

$$MCC = \frac{c \times s - \sum_k p_k \times t_k}{\sqrt{(s^2 - \sum_k p_k^2) \times (s^2 - \sum_k t_k^2)}} \tag{1}$$

where c is the total number of correctly classified samples, s is the total number of samples, p_k is the number of times class k was predicted, and t_k is the number of times class k truly occurred. The coefficient yields values in the interval [-1, 1], with 1 indicating perfect prediction, 0 signifying random prediction, and -1 denoting complete misclassification. The MCC takes into account all elements of the confusion matrix, providing a more comprehensive evaluation than metrics such as accuracy or F1-score, especially for imbalanced datasets. The MCC also remains informative even when class sizes differ significantly and is sensitive to both over-prediction and under-prediction of classes.

We use the scikit-learn implementation of the MCC for our evaluation, which is available in the module:

```
sklearn.metrics.matthews_corrcoef
```

E Accuracy-based Evaluation of the Benchmarks

Table 6 shows the 10-fold mean accuracy (%) scores of the best performing variants of the Enformer [Avsec et al., 2021], DNABERT [Zhou et al., 2023], Nucleotide Transformer v2 [Dalla-Torre et al., 2023], and HyenaDNA [Nguyen et al., 2023] on the Nucleotide Transformer (NT) benchmarks. The scores are sourced from a leaderboard maintained by the authors of [Dalla-Torre et al., 2023] on the Hugging Face platform [InstaDeepAI, 2023].

**Genomic Benchmark**	CNN	DNABERT	GPT	HyenaDNA [Nguyen et al., 2023]	ENBED (no pre-training)	ENBED
Mouse Enhancers	69.0	66.9	80.1	<u>85.1</u>	75.5	**90.3**
Human Enhancers (Cohn)	69.5	<u>74.0</u>	70.5	**74.2**	54.3	71.2
Human Enhancers (Ensembl)	68.9	85.7	83.5	<u>89.2</u>	83.3	**92.2**
Coding vs Intergenomic	87.6	<u>92.5</u>	88.8	91.3	84.2	**93.0**
Human vs Worm	93.0	96.5	95.6	<u>96.6</u>	90.8	**97.3**
Human Regulatory Elements	<u>93.3</u>	88.1	91.5	**93.8**	80.8	90.2
Human Promoter (Non-TATA)	84.6	85.6	87.7	<u>96.6</u>	83.4	**97.2**
Human OCR (Ensembl)	68.0	75.1	73.0	<u>80.9</u>	64.3	**81.9**

Table 5: **Genomic Benchmarks.** Accuracy (%) scores of the **best** and <u>second-best</u> model in the Genomic Benchmarks datasets [Grevsova et al., 2022]. The baseline CNN and GPT scores was calculated by the authors of [Grevsova et al., 2022] and [Nguyen et al., 2023] respectively.

References

Žiga Avsec, Vikram Agarwal, Daniel Visentin, et al. Effective gene expression prediction from sequence by integrating long-range interactions. *Nature methods*, 18(10):1196–1203, 2021.

Hugo Dalla-Torre, Liam Gonzalez, Javier Mendoza Revilla, Nicolas Lopez Carranza, Adam Henryk Grzywaczewski, Francesco Oteri, Christian Dallago, Evan Trop, Hassan Sirelkhatim, Guillaume Richard, Marcin J. Skwark, Karim Beguir, Marie Lopez, and Thomas Pierrot. The nucleotide transformer: Building and evaluating robust foundation models for human genomics. *bioRxiv*, 2023. URL `https://api.semanticscholar.org/CorpusID:255943445`.

Moritz Gerstung, Christian Beisel, Markus Rechsteiner, Peter Wild, Peter Schraml, Holger Moch, and Niko Beerenwinkel. Reliable detection of subclonal single-nucleotide variants in tumour cell populations. *Nature communications*, 3(1):811, 2012.

Katarina Grevsova, Vlastimil Martinek, David Cechak, Petr Simecek, and Panagiotis Alexiou. Genomic benchmarks: a collection of datasets for genomic sequence classification. *BMC Genomic Data*, 24, 2022. URL `https://api.semanticscholar.org/CorpusID:249649488`.

InstaDeepAI. Nucleotide transformer benchmark. *Hugging Face*, Sep 2023. hugging-face.co/spaces/InstaDeepAI /nucleotide_transformer_benchmark.

Eric D Nguyen, Michael Poli, Marjan Faizi, Armin W. Thomas, Callum Jacob Birch-sykes, Michael Wornow, Aman Patel, Clayton M. Rabideau, Stefano Massaroli, Yoshua Bengio, Stefano Ermon, Stephen A. Baccus, and Christopher Ré. Hyenadna: Long-range genomic sequence modeling at single nucleotide resolution. *ArXiv*, 2023. URL `https://api.semanticscholar.org/CorpusID:259274952`.

Raul Rabadan, Gyan Bhanot, Sonia Marsilio, Nicholas Chiorazzi, Laura Pasqualucci, and Hossein Khiabanian. On statistical modeling of sequencing noise in high depth data to assess tumor evolution. *Journal*

**NT Benchmark**	Enformer	DNABERT-2	NT (v2)	HyenaDNA	ENBED (no pre-training)	ENBED
H3	85.9	89.3	<u>89.5</u>	88.9	64.4	**90.6**
H3K14ac	63.5	75.9	76.9	<u>80.9</u>	51.6	**81.4**
H3K36me3	67.1	79.7	<u>81.3</u>	80.8	61.1	**82.7**
H3K4me1	64.6	75.8	<u>77.7</u>	75.8	58.4	**77.9**
H3K4me2	63.0	68.0	67.6	<u>73.9</u>	55.9	**75.7**
H3K4me3	56.5	67.3	69.5	<u>77.5</u>	50.9	**77.9**
H3K79me3	74.7	80.7	81.3	<u>83.7</u>	83.1	**85.4**
H3K9ac	70.8	77.1	78.0	<u>79.3</u>	60.2	**82.6**
H4	86.6	89.9	<u>90.5</u>	88.2	74.3	**91.8**
H4ac	63.8	73.1	74.9	<u>78.4</u>	67.2	**80.5**
Promotor (all)	95.4	97.1	<u>97.6</u>	96.0	94.3	**98.0**
Promotor (non-TATA)	95.5	97.2	<u>97.6</u>	96.0	94.4	**98.0**
Promotor (TATA)	96.0	95.5	<u>96.6</u>	94.1	92.9	**96.8**
Splice acceptor	91.4	<u>97.5</u>	**98.7**	95.8	87.8	95.8
Splice donor	90.6	<u>96.3</u>	**98.7**	95.8	87.7	95.4
Enhancer	72.3	75.7	<u>77.3</u>	75.9	65.2	**78.3**
Enhancer Types	55.4	62.0	<u>62.6</u>	59.5	51.4	**70.0**

Table 6: **Nucleotide Transformer (NT) Benchmarks.**

of Statistical Physics, 172(1):143155, December 2017. ISSN 1572-9613. doi: 10.1007/s10955-017-1945-1. URL `http://dx.doi.org/10.1007/s10955-017-1945-1`.

Zhihan Zhou, Yanrong Ji, Weijian Li, Pratik Dutta, Ramana Davuluri, and Han Liu. Dnabert-2: Efficient foundation model and benchmark for multi-species genome, 2023.
